# 641. Real-Time Whole Genome Sequencing Surveillance of Healthcare Associated SARS-CoV-2, Respiratory Syncytial Virus (RSV) and Influenza

**DOI:** 10.1093/ofid/ofae631.206

**Published:** 2025-01-29

**Authors:** Vatsala Rangachar Srinivasa, Marrisa P Griffifth, Rose Patrick, Kady D Waggle, Dumebi Okonkwo, Daria Van Tyne, Graham M Snyder, Alexander Sundermann, Lora Pless, Lee Harrison

**Affiliations:** University of Pittsburgh, Pittsburgh, PA; University of Pittsburgh, Pittsburgh, PA; University of Pittsburgh, Pittsburgh, PA; University of Pittsburgh, Pittsburgh, PA; University of Pittsburgh, Pittsburgh, PA; University of Pittsburgh School of Medicine, Pittsburgh, Pennsylvania; University of Pittsburgh, Pittsburgh, PA; University of Pittsburgh, Pittsburgh, PA; University of Pittsburgh, Pittsburgh, PA; University of Pittsburgh, Pittsburgh, PA

## Abstract

**Background:**

Seasonal respiratory viruses contribute to substantial healthcare burden, marked by elevated rates of hospitalizations, morbidity and mortality. The transmission dynamics of respiratory viruses in adult acute care hospitals are not well described. To address this, we performed whole genome sequencing (WGS) surveillance to investigate transmission of SARS-CoV-2, RSV, and influenza among hospitalized patients in a single health system.

**Figure:**
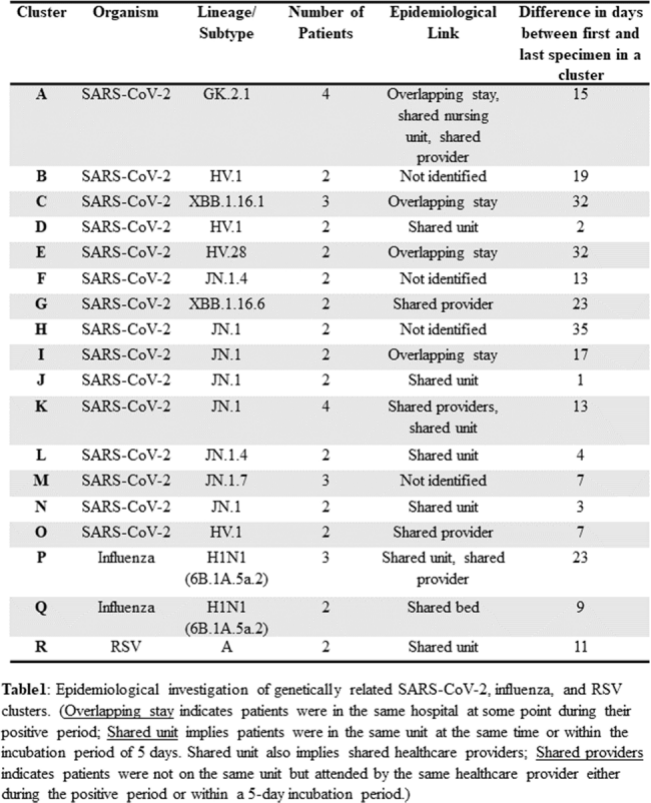

**Methods:**

From August 30, 2023 to March 31, 2024, nasal swab specimens positive for SARS-CoV-2, influenza, or RSV were collected weekly from inpatients hospitalized for ≥ 3 days from two hospitals. Additional specimens collected from the same patient within a 14-day period were excluded. Specimens with qPCR cycle threshold (Ct) values ≥ 34, ≥ 39, and ≥ 31 for SARS-CoV-2, RSV and influenza, respectively, were excluded from WGS. WGS was performed using tiled PCR amplicons or Twist Bioscience hybridization method, followed by bioinformatic analyses to assess genetic relatedness [≤ 2 single nucleotide polymorphisms (SNPs) except for influenza (≤ 3 SNPs)]. Clusters were further investigated for epidemiological connections (shared locations or staff).

**Figure:**
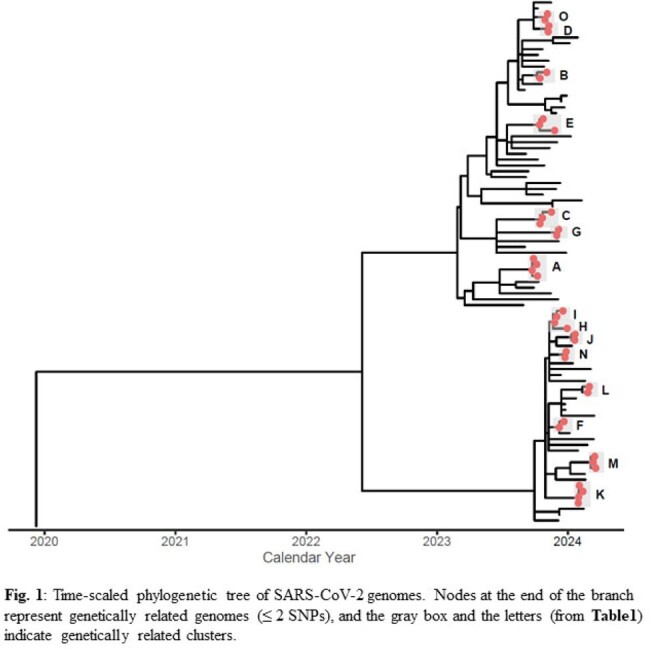

**Results:**

We collected 179 SARS-CoV-2, 37 influenza and 16 RSV specimens, of which 93 specimens did not meet the inclusion and the qPCR QC criteria. We sequenced 84% (87/104) of SARS-CoV-2, 86% (19/22) of influenza, and 92% (12/13) of RSV specimens. Of these, 45% (39/87) of SARS-CoV-2 and 32% (6/19) of influenza genomes formed genetically related clusters, each with 2-4 patients. We identified an epidemiological link for 73% (11/15) of SARS-CoV-2 and 100% (2/2) of influenza clusters (**Fig. 1 & Fig. 2**). For RSV genomes, we identified one cluster with two epidemiologically linked patients (**Table1**). On average, the transmission clusters spanned a duration of 15 days (range: 1−35 days, **Table1**).

**Figure:**
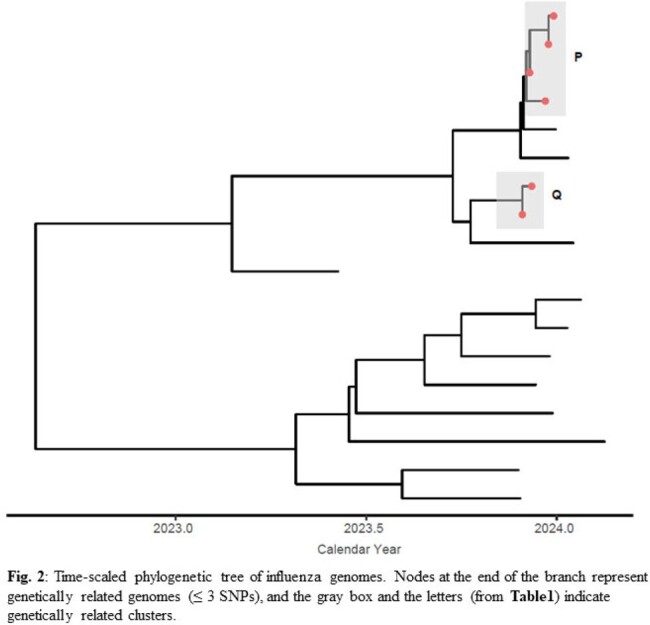

**Conclusion:**

Respiratory viral transmission is frequent in hospitals, with around 40% of sequenced specimens genetically related to each other. While this study serves as an initial step towards possible implementation of real-time WGS surveillance, further investigation is needed to address broader implications, including implementation of infection prevention strategies to address transmission clusters.

**Disclosures:**

**Graham M. Snyder, MD, SM**, Infectious Diseases Connect: Advisor/Consultant **Alexander Sundermann, DrPH, CIC, FAPIC**, OpGen: Honoraria **Lee Harrison, MD**, GSK: Advisor/Consultant|Merck: Advisor/Consultant|Pfizer: Advisor/Consultant|Sanofi: Advisor/Consultant

